# The relative patient costs and availability of dental services, materials and equipment in public oral care facilities in Tanzania

**DOI:** 10.1186/s12903-015-0061-3

**Published:** 2015-07-01

**Authors:** Kasusu K. Nyamuryekung’e, Satu M. Lahti, Risto J. Tuominen

**Affiliations:** Department of Orthodontics, Paedodontics and Community Dentistry, School of Dentistry, Muhimbili University of Health and Allied Sciences, Dar es Salaam, Tanzania; Department of Community Dentistry, University of Turku, Turku, Finland; Department of Public Health, University of Turku and Hospital District of Southwest Finland, Turku, Finland

**Keywords:** Cost, Dental services, Equipment availability, Material availability, Patient charges

## Abstract

**Background:**

Patient charges and availability of dental services influence utilization of dental services. There is little available information on the cost of dental services and availability of materials and equipment in public dental facilities in Africa. This study aimed to determine the relative cost and availability of dental services, materials and equipment in public oral care facilities in Tanzania. The local factors affecting availability were also studied.

**Methods:**

A survey of all district and regional dental clinics in selected regions was conducted in 2014. A total of 28/30 facilities participated in the study. A structured interview was undertaken amongst practitioners and clinic managers within the facilities. Daily resources for consumption (DRC) were used for estimation of patients’ relative cost. DRC are the quantified average financial resources required for an adult Tanzanian’s overall consumption per day.

**Results:**

Tooth extractions were found to cost four times the DRC whereas restorations were 9–10 times the DRC. Studied facilities provided tooth extractions (100 %), scaling (86 %), fillings (79 %), root canal treatment (46 %) and fabrication of removable partial dentures (32 %). The ratio of tooth fillings to extractions in the facilities was 1:16. Less than 50 % of the facilities had any of the investigated dental materials consistently available throughout the year, and just three facilities had all the investigated equipment functional and in use.

**Conclusions:**

Dental materials and equipment availability, skills of the practitioners and the cost of services all play major roles in provision and utilization of comprehensive oral care. These factors are likely to be interlinked and should be taken into consideration when studying any of the factors individually.

**Electronic supplementary material:**

The online version of this article (doi:10.1186/s12903-015-0061-3) contains supplementary material, which is available to authorized users.

## Background

Low and middle income countries (LMICs) comprise 84 % of the global population, experience 90 % of the global disease burden and are mostly found in sub-Saharan Africa and South East Asia [1]. Many of these countries are also undergoing a disease-epidemiology transition, leading to increased incidence of chronic diseases in addition to the already prevalent infectious diseases [2]. In light of these challenges, a robust and a responsive health system is vital for maintenance and provision of heath care. A balanced ratio of health care providers to population, facilities, equipment and supplies is essential for effective functioning of any health care system [3]. In many instances access to quality health and oral health care is limited in LMICs. Health services have been reported as frequently unavailable, prohibitively expensive, underfunded, under staffed or under equipped [4]. Generally, information on the availability of dental services and their prices in LMICs is limited.

Affordability and accessibility are important determinants of health service utilization in many LMICs. Nonetheless, most of the countries depend on direct out-of-pocket expenditure as the primary source of health system finance [1, 5, 6]. Furthermore, it has been suggested that in many LMICs, oral health services are in worse state than general health services [7, 8]. The causes for this are varied, ranging from low prioritization in health policies to a shortage of oral health care providers and facilities [4, 7]. In Africa as a whole, the dentist-to-population ratio is approximately 1:150,000 [8]; in Tanzania it is 1:360,000 [9]. WHO states that in industrialized countries, the dentist-to-population ration is 1:2000 [8].

Tanzania is a large East African country with a population of 45 million. More than 60 % of its population consists of subsistence farmers and about a quarter are living in poverty [10, 11]. Like many countries in Africa, it has a cost-sharing policy, whereby patients are required to pay for their health care. This approach has been suggested as both a barrier to accessing quality health care and a contributor to poverty for large population groups [12, 13]. Despite attempts to increase the number of people enrolled in available health insurance schemes, by 2012 only 11.3 % of the population had such cover [14] with the majority of the uninsured being the poor and disadvantaged [15]. It has been reported that a significant proportion of poor Tanzanian households have experienced such high level of health expenditures as to risk impoverishment [13].

Oral pain is the main cause of attendance at all public dental clinics in Tanzania [16]. A nationally representative survey in 2007 revealed that more than half of the population suffered from oral pain or discomfort within twelve months preceding the survey. However, only a quarter of those who experienced oral pain sought oral care, most commonly because of financial constraints [16]. The most common oral care carried out in public dental clinics is tooth extractions [17, 18]. The limited number of clinics offering restorative care in Tanzania has been implicated as an obstacle towards accessing restorative services when warranted [18]; other barriers to restorative services include faulty equipment, lack of funds to purchase materials and dental materials not being available [19].

The aim of this study was to determine the relative patient costs and availability of dental services, materials and equipment in public oral care facilities in Tanzania. The effect of budgetary sufficiency, reliability of acquisition and timeliness of dental materials delivery on availability of dental materials was considered.

## Methods

### Setting

Tanzania is divided into six geographical zones; five in the mainland and one in the isles. Each zone comprises a cluster of regions. Four of the zones in the mainland have designated zonal referral hospitals within one of their regions, situated in the regions of Mbeya, Mwanza, Kilimanjaro and Dar es Salaam.

Regions are further divided into several districts. Public dental clinics are placed in administrative headquarters within the district and regional hospitals.

### Sampling

All district and regional hospitals (*n* = 30) and dental practitioners working within these facilities in the regions of Mbeya, Mwanza, Kilimanjaro and Dar es Salaam were invited to participate in the study.

### The survey

This study was conducted using face-to-face structured interviews. The interviewer was a dental professional, and interviews were carried out among clinic managers of the hospitals and dental practitioners working within these facilities in a hospital setting. Dental surgeons, assistant dental officers and dental therapists are the certified dental personnel in Tanzania. However, due to their limited number, other cadres and paraprofessionals may be given basic training in oral care to enable them to provide dental services. For the analyses, practitioners identified as dental surgeons and assistant dental officers were categorized as “Dental officers”. Dental therapists, dental auxiliaries, health attendants, clinical and assistant medical officers providing oral care in the facilities were categorized as “Other cadres”.

### Development of the questionnaire

No study tool was available to be used directly in this research setting. Therefore, we developed a questionnaire in the local language, Kiswahili. Two questionnaires were developed: for the clinic managers (Additional file 1) and practitioners (Additional file 2) in the clinic.

A pilot assessment was conducted to determine dental services most commonly offered in Dar es Salaam. These were identified as: tooth extractions, fillings, root canal treatment, scaling and fabrication of removable dentures. Questions on provider competency assessed tooth extractions and fillings, as these were the most frequently offered services.

Basic dental materials and equipment required in public oral care facilities were identified from the central oral health unit of the Ministry of Health, Dar es Salaam. Local factors that could affect availability of dental materials were elucidated from a previous survey conducted amongst dental practitioners in Tanzania [19]. These factors were subsequently re-categorized and included in the questionnaire as: budgetary sufficiency, reliability of dental material acquisition, and timeliness of delivery.

The feasibility of the questionnaire was assessed by field-testing in dental facilities within a region not involved in the study. Adjustments in wording and measurements of variables were made accordingly.

The final questionnaires encompassed matters related to types of services offered and their price, material and equipment availability, local structural factors affecting material availability, treatment profiles of the clinic, and practitioners’ self-perceived competency for provision of selected dental services.

### Measurements

The price for extractions and single surface, one-tooth fillings for out of pocket payments (OOP) from the facilities were reported in Tanzanian shillings (Tshs). A large proportion of Tanzanians are informally employed, without steady monthly income. Therefore, to contextualize the magnitude of OOP prices, the Tanzanian index amount of resources required for basic consumption was used as a reference for service affordability [11].

The average monthly financial resources required in Tanzania for basic consumption is Tshs 36,482 per adult, making the daily resources required for basic consumption (DRC) 1, 216 Tshs (1 US$ = 1658.35 Tshs, July 2014). Out of pocket payments were presented as the proportional share of the prices of dental services from an adult’s basic daily financial resources required for overall consumption (the relative cost, RC) using the following formula:

$$ \mathrm{R}\mathrm{C} = \mathrm{O}\mathrm{O}\mathrm{P}/\mathrm{D}\mathrm{R}\mathrm{C} $$

where OOP is the price of a particular service in Tshs; DRC is 1, 216 Tshs.

The provision of tooth extractions, fillings, scaling, root canal treatment and fabrication of removable partial dentures was investigated by asking with a *yes/no* response: “*Do you offer the following services at your clinic?*” To describe the treatment profile of the clinic, we used registers containing the list of extraction and filling procedures performed in the facility during the last 3 months.

The availability and functional status of selected dental equipment was investigated by asking *“Do you have the following dental equipment in your facility and what is its status?*” The response alternatives were: *not available, not in use (faulty), not in use (functional), in use (faulty)* and *in use (fully functional).* The equipment considered were: dental radiography units, autoclaves, amalgamators, composite light cure machines, hand pieces, compressors and dental chairs.

Availability of dental materials (amalgam, composite, zinc oxide eugenol, glass ionomer cement and calcium hydroxide) and local structural factors affecting their availability (budgetary sufficiency, reliability, and timeliness in obtaining materials) were investigated using a numeric rating scale (NRS) with endpoints 0 (worst possible situation) to 10 (best possible situation). The segment extremes were; for material availability *“never available” (0)* and *“always available” (10)*; for budgetary sufficiency *“highly insufficient” (0)* and *“highly sufficient” (10)*; for reliability *“highly unreliable” (0) “highly reliable” (10)*; and for timeliness *“never delivered on time” (0)* and *“always delivered on time” (10)* during the last year. The scores for local structural factors were subsequently categorized into “poor” (0–3), “moderate” (4–6) and “good” (7–10).

Self-rated competence assessment for performing selected dental services (tooth extractions and tooth restorations using dental amalgam, composite, glass ionomer cement, zinc oxide and calcium hydroxide) was also determined using NRS where the extremes were *“not competent at all” (0)* and *“extremely competent” (10).*

### Statistical methods

The differences in practitioner groups’ mean perceived competencies scores were tested using Student’s independent sample t-test. P-values less than 0.05 were considered statistically significant. Statistical analyses were done using SPSS, version 21.

### Ethical considerations

Written approval for this study was obtained from the Ethical Committee of the Muhimbili University of Health and Allied Sciences (MU/DRP/AEC/Vol. XVIII/118). Informed consent was also obtained from all the subjects who participated in this study.

## Results

Of all the invited facilities, two (2) could not be reached. Therefore, a total of 28 facilities (Mbeya: nine; Kilimanjaro: six; Mwanza: seven; Dar es Salaam: six) and 56 practitioners participated in this study. Tooth extractions were found to cost patients an average of four-times their DRC. Restorations were on average 9–10 times the DRC. The difference in prices of various services varied approximately 10 fold across facilities (Table 1). Tooth extractions were provided by all studied facilities, scaling by 86 %, fillings by 79 %, root canal treatment by 46.4 % and fabrication of removable partial dentures by 32 %. Despite more than three-quarters of the facilities reporting provision of tooth filling services, they constituted a very small proportion of overall treatment. The treatment profile revealed a ratio of 1:16 for tooth fillings to extractions.

**Table 1:** Variability of relative costs (RC; the ratio of patient charges to the resources required for one day of basic consumption) for dental services

	Valid	Missing	Median RC	Minimum RC	Maximum RC
Dental consultation	28	0	2.47	0.82	8.22
Tooth extraction	28	0	4.11	0.82	8.22
Amalgam restoration	23	5	8.22	2.47	24.67
Glass ionomer cement restoration	24	4	8.22	2.47	24.67
Composite restoration	23	5	8.22	2.47	32.89

The distribution of availability of dental materials was largely bimodal, with highest frequencies observed on two extremes of availability spectrum. Zinc oxide eugenol was most and composite least frequently available (Table 2).

**Table 2:** Relative frequency distribution and median availability of materials in dental facilities (*n* = 28) within last one year

	Never available (% of clinics)	Median availability score (range)	Always available (% of clinics)
Amalgam	28.6	5.5 (0, 10)	42.9
Composite	39.3	5.5 (0, 10)	42.9
Glass ionomer cement	32.1	6.0 (0, 10)	35.7
Zinc Oxide Eugenol	21.4	8.3 (0,10)	42.9
Calcium Hydroxide	35.7	7.5 (0, 10)	39.3

Dental radiography units were not available in half of all studied facilities, and only a quarter of facilities had fully functional units. Slightly less than half of the facilities either do not possess or have broken dental autoclaves. Equipment that was most frequently reported to be available and in use was: dental chairs, compressors and light cure machines (Table 3). One facility had none and two did not have at least four of the investigated equipment; three facilities had all the equipment fully functional and in use.

**Table 3:** Percentage distribution of availability and functional status of dental equipment in dental facilities (*n* = 28)

	Not available	Not in use, faulty	Not in use, functional	In use, faulty	In use, fully functional
Dental radiograph	53.6	7.1	10.7	3.6	25.0
Autoclave	28.6	14.3	0.0	7.1	50.0
Amalgamator	25.0	3.6	21.4	7.1	42.9
Light cure machine	25.0	3.6	10.7	0.0	60.7
Hand piece	14.3	7.1	7.1	17.9	53.6
Compressor	10.7	7.1	10.7	7.1	64.3
Dental chair	3.6	0.0	0.0	28.6	67.9

Timeliness in delivery of dental materials was rated as moderate-good by 36 % of the facilities. Budgetary sufficiency and reliability in acquiring dental materials was rated as moderate-good by 71 and 64 % of the facilities, respectively. Of the local structural factors that affect dental material availability; timeliness in delivery was rated most poorly, followed by reliability and then budgetary sufficiency for material procurement (Table 4).

**Table 4:** Percentage distribution and median scores of local structural factors affecting dental material availability (Interquartile range in parentheses)

	Poor (0–3)	Moderate (4–6)	Good (7–10)	Median score (IQR)
Reliability acquiring	35.7	17.9	46.4	5.5 (0,10)
Budgetary sufficiency	28.6	32.1	39.3	4.5 (0,10)
Timeliness in delivery	64.3	10.7	25.0	0.0 (0,10)

Dental officers generally had higher perceived levels of skills compared to other cadres working in the dental clinic. Tooth extractions were the only service whereby the skill level was perceived similarly between the two groups (Table 5).

**Table 5:** Mean perceived competency scores (95 % CI in parentheses) of dental officers and other cadres for different treatment options in facilities (p value for t-test)

	Dental officers (*n* = 29)	Other cadres (*n* = 27)	p value
Extractions	9.7 (9.4, 9.9)	9.1 (8.3, 9.9)	0.187
Amalgam restorations	9.0 (8.5, 9.6)	6.5 (5.2, 7.8)	0.001
Composite restorations	9.1 (8.7, 9.6)	6.7 (5.4, 8.1)	0.001
Glass ionomer cement restorations	9.3 (9.0, 9.7)	7.4 (6.1, 8.8)	0.005
Zinc oxide restorations	9.8 (9.6, 10.0)	8.1 (6.7, 9.5)	0.05
Calcium hydroxide application	9.5 (9.0, 9.9)	6.4 (4.8, 7.9)	0.001

## Discussion

Although many facilities did not offer restorative services, all offered tooth extraction services. Consequently, tooth extractions were the most common treatment provided. The cost for all dental services was generally high. In addition, dental materials were sporadically available and a full set of functional equipment was rarely present in the facilities.

Cost of services is an important determinant of utilization of health services [20, 21]. This study revealed that up to 50 % of Tanzanians would need to invest financial resources equivalent to 9 days-worth of consumption [11] in order to pay for a filling, illustrating the high cost of dental care. Previous studies in Tanzania, Burkina Faso and Nigeria have indicated cost, and specifically the price of services, as one of the major barriers towards utilization of oral care services [12, 22–25]. In Benin City, Nigeria, even among respondents with high socioeconomic status, 12 % were unable to afford dental treatment when required [26].

In our study, the price for dental services was shown to be highly variable across facilities. This may be due to differences in prices of services between regional and district hospitals, as they function at different hierarchical levels in the Tanzanian health system [27]. Nonetheless, even when assessed separately, this variation persisted within regional and district facilities. Therefore, it is probable that dental facilities have their prices set independently, leading to observed variations. This variability might selectively preclude consumers with the same socioeconomic status across different geographic locations from accessing oral care. None of the previous studies [12, 22–25] have determined the price of dental services directly from the facilities, relying instead on reports from the served population. Our findings confirm the conclusions of previous studies, that the costs for dental services are considerably high.

Due to annual or even seasonal fluctuations in population income levels, the national index of monthly financial resources for basic consumption was considered a valid proxy for purchasing power. Besides, since this index is based on the consumption patterns of the poorest 10–50 %, it allows for an assessment of the financial barrier towards utilization of dental services among the poorest [11]. Utilization of dental care is reported to be dependent on ability to pay, rather than on need of care [8, 20, 28]. Bearing in mind that about a quarter of the Tanzanian population are living in poverty [10] with many competing needs and interests, it is likely that many would postpone utilization of dental care. Whenever they do use these services, most consumers may be expected to opt for extraction, because of the relatively smaller resource allocation required than for restorative work.

Dental materials and equipment availability are necessary for delivery of comprehensive oral care [4]. In line with previous studies conducted in Eastern Africa (Tanzania, Kenya, Sudan) this study provided further evidence of widespread shortage of dental materials and equipment in public facilities [19, 25, 29, 30]. The finding that less than half of the facilities had any of the materials consistently throughout the previous year means that restorative services are not available in many facilities, even if demanded by the dental patients. Additionally, the proportion of facilities that offer restorative services throughout the year is likely to be smaller than currently reported due to intermittent availability of dental materials. The government-endorsed supplier for public hospitals in Tanzania does not stock dental materials or equipment [31]. Consequently, dental facilities throughout the country are forced to rely mostly on private, local vendors for their supplies. Nevertheless, timeliness of material delivery in most facilities was rated poorly, suggesting erratic and lengthy waiting times for the supply of materials. However, budgetary sufficiency for procurement of dental materials was favourably rated, indicating readily available financial resources. Therefore, it appears as if either the supply of dental materials from private vendors fails to meet demand or that procurement processes are inefficient, leading to undue delays in obtainment of dental materials and equipment in facilities.

It is possible that equipment availability influences material availability. Essentially, a facility does not have an incentive to purchase materials if there is no equipment to work with, and vice versa. However, due to the cross sectional nature of the study and the sample size involved, it was not possible to establish conclusively a causal relationship between these factors. Furthermore, it should be noted that these findings are from facilities within regions with zonal referral hospitals. These regions probably have better health care infrastructure compared to other regions in the country. Therefore these findings may be considered to give an optimistic view of the overall situation.

There is a tendency for predominantly symptom-oriented attendance for dental care in developing countries in Africa. The major reason for dental attendance in public facilities is due to toothache as a result of dental caries [16, 29, 32, 33]. Treatment profiles for the vast majority of the facilities in sub Saharan Africa are highly skewed in favour of extractions as compared to fillings [29, 33–36], similar to our findings. In accordance with previous studies indicating a high degree of satisfaction with tooth extraction services amongst the Tanzanian population [32, 37], practitioners correspondingly reported highest competencies in this skill. However, when compared to dental officers, other cadres working in dental facilities reported systematically lower restorative competencies. This is likely due to “other cadres” containing practitioners without formal training in dental care (clinical officers and assistant medical officers), or with very basic dental training that focuses on emergency dental care (dental therapists and dental auxiliaries). The perceived ability of practitioners to perform a clinical procedure is an important determinant of the kind of treatment offered to patients [38, 39]. Hence, this treatment profile is possibly a reflection of training curriculum that places heavy emphasis on tooth extractions [40] or an indication that these practitioners operate in an environment where extractions are the mainstay treatment offered due to limited treatment options. This is corroborated by our findings indicating that many of the facilities do not have materials, equipment or the skilled workforce to provide restorations.

All of the interviews were conducted by a dental practitioner, and the respondents were aware of this. However, it is not expected that this might have led to any bias in responding. On the contrary, it is expected that an interview with a colleague might have encouraged them to be more forthcoming with their responses. Due to the limited numbers of both the facilities and responding practitioners, further detailed analyses were not possible. As a result, there remain many potential areas of interactions that remain unexplored. Additionally, not all practitioners employed in the facilities were interviewed; only those that were available on the day of the interview were included. This may act as a potential source of bias in this study. Nonetheless, the observed absenteeism is unlikely to be due to this particular research work, as the facilities were not informed in advance. Larger samples and repeated measurements, as well as inclusion of more regions will increase representativeness, as well as allow for a more thorough analyses of factors associated with cost and availability of materials and equipment. It may be interesting to conduct further studies to determine the effective availability of dental care professionals in their respective facilities and the regional density of private local vendors for dental materials and their effect on availability of dental materials and equipment in facilities.

## Conclusions

Dental materials and equipment availability, skills of the practitioners and the cost of services all play major roles towards provision and utilization of comprehensive oral care. It is likely that these factors are all interlinked and should be taken into consideration when studying any of the factors individually. It is possible that secondary factors such as procurement processes are important in this setting when describing patterns of service provision, although further research would be required to investigate these factors.

## Additional files

Additional file 1:
**Questionnaire for dental facilities' managers.**
Additional file 2:
**Questionnaire for practitioners in the dental clinic.**
