# Supplementary material for: The relative patient costs and availability of dental services, materials and equipment in public oral care facilities in Tanzania
Source: BMC Oral Health. 2015 Jul 1;15:74. doi: 10.1186/s12903-015-0061-3 (PMC4487056; doi:10.1186/s12903-015-0061-3)
Supplement: Additional file 1: — Questionnaire for dental facilities' managers. [file 12903_2015_61_MOESM1_ESM.docx]

**Questionnaire for dental facilities**

SN: ___________________________ Region: ________________________

District: _______________________ Hospital Name: ______________________

1. Professionals available in your facility:

**Qualifications** **Number**

1. Dental Therapist __________
2. Laboratory Technician __________
3. Assistant Dental Officer __________
4. Doctor of Dental Surgery __________
5. Health attendant __________
6. Specialist:_____________ __________
7. Others: ________________
8. How many patients were seen in this facility within the last 3 months? ______________ (verify from attendance roster?)
9. What are the acceptable modes of payment for dental services in your facility?
   1. Out of pocket 1. Yes 2. No
   2. National Health Insurance Fund (NHIF) 1. Yes 2. No
   3. TIKA (Tiba kwa Kadi) 1. Yes 2. No
   4. CHF (Community Health Fund) 1. Yes 2. No
   5. Others: _________________________
10. How many patients utilized the following modes for payment of services? Within the last 3 months? (verify from roster)
11. Out of pocket _____________
12. NHIF _____________
13. TIKA _____________
14. CHF _____________
15. Others _____________
16. Does your clinic offer any dental restorative services?

Yes

No

1. What restorative services do you offer in your clinic?

| **Restorative service offered** |  |  |
| --- | --- | --- |
| 1. Tooth filling | **Yes (1)** | **No (2)** |
| 1. Scaling and root planning | **Yes (1)** | **No (2)** |
| 1. Root canal treatment | **Yes (1)** | **No (2)** |
| 1. Fabrication of removable dentures | **Yes (1)** | **No (2)** |
| 1. Fabrication of fixed dentures | **Yes (1)** | **No (2)** |
| 1. Others:_____________________________ | **Yes (1)** | **No (2)** |

1. Who is your supplier of dental materials? _____________________________________________________________________
2. On a scale of **0-10**, where **“0”** is **“never available”** and **“10”** is **“always available”**, how do you rate the availability of the following dental materials in your clinic in the last one year?

| **Dental Material** | **Availability** |
| --- | --- |
| 1. Dental amalgam |  |
| 1. Conventional resin composite (light cure) |  |
| 1. Packable resin composite (cold cure) |  |
| 1. Glass Ionomer cement |  |
| 1. Zinc Oxide/ Zinc phosphate |  |
| 1. Calcium hydroxide |  |

1. How many people have attended your clinic within the last 3 months for extractions and restorations using the following materials? (Verify from roster)

| **Dental Material** | **Number of patients** |
| --- | --- |
|  |  |
| 1. Tooth extractions |  |
| 1. Dental amalgam |  |
| 1. Conventional resin composite (light cure) |  |
| 1. Packable resin composite (cold cure) |  |
| 1. Glass Ionomer cement |  |
| 1. Zinc Oxide/ Zinc phosphate |  |

1. What is the price of the following services in Tshs (single surface, one tooth) using the following materials and payment schemes?

| **Dental Material** | **Out of pocket payment (Tshs)** | **Insurance (Tshs)** | | |
| --- | --- | --- | --- | --- |
|  |  | **NHIF** | **TIKA** | **CHF** |
| 1. Dental consultation |  |  |  |  |
| 1. Tooth extraction |  |  |  |  |
| 1. Dental amalgam |  |  |  |  |
| 1. Conventional resin composite (light cure) |  |  |  |  |
| 1. Packable resin composite (cold cure) |  |  |  |  |
| 1. Glass Ionomer cement |  |  |  |  |
| 1. Zinc Oxide/ Zinc phosphate |  |  |  |  |

1. On a scale of **0-10**, whereby **“0”** is **“highly insufficient”** and **“10”** is **“highly sufficient”**, how do you rate the sufficiency of the budget for procuring dental materials in your facility in the last one year?___________
2. On a scale of **0-10**, whereby **“0”** is **“highly unreliable”** and **“10”** is **“highly reliable”**, how do you rate the reliability of supply of dental materials in your facility in the last one year? _______________
3. On a scale of **0-10**, whereby **“0”** is **“never delivered on time”** and **“10”** is **“always delivered on time”**, how do you rate the timeliness of the delivery of ordered dental materials? ______________________
4. Do you have the following instruments in your facility?

| **Dental Instrument** | **Not available (1)** | **Not in use, faulty (2)** | **Not in use, functional (3)** | | **In use, faulty (4)** | **In use, fully functional (5)** |
| --- | --- | --- | --- | --- | --- | --- |
| 1. Amalgamator | **(1)** | **(2)** | **(3)** | | **(4)** | **(5)** |
| 1. Light cure machine | **(1)** | **(2)** | **(3)** | | **(4)** | **(5)** |
| 1. Hand piece | **(1)** | **(2)** | **(3)** | | **(4)** | **(5)** |
| 1. Compressor | **(1)** | **(2)** | **(3)** | | **(4)** | **(5)** |
| 1. Dental chair | **(1)** | **(2)** | **(3)** | | **(4)** | **(5)** |
| 1. Dental x-ray unit | **(1)** | **(2)** | **(3)** | | **(4)** | **(5)** |
| 1. Autoclave | **(1)** | **(2)** | **(3)** | | **(4)** | **(5)** |
| 1. Tweezers | **Available (1)** | | | **Not available (2)** | | |
| 1. Mixing spatula | **Available (1)** | | | **Not available (2)** | | |
| 1. Amalgam carver | **Available (1)** | | | **Not available (2)** | | |
| 1. Amalgam carrier | **Available (1)** | | | **Not available (2)** | | |
| 1. Condenser | **Available (1)** | | | **Not available (2)** | | |
| 1. Plastic filling instrument | **Available (1)** | | | **Not available (2)** | | |
| 1. Mouth mirrors | **Available (1)** | | | **Not available (2)** | | |
| 1. Probes | **Available (1)** | | | **Not available (2)** | | |
| 1. Excavators | **Available (1)** | | | **Not available (2)** | | |
