# Supplementary material for: The relative patient costs and availability of dental services, materials and equipment in public oral care facilities in Tanzania
Source: BMC Oral Health. 2015 Jul 1;15:74. doi: 10.1186/s12903-015-0061-3 (PMC4487056; doi:10.1186/s12903-015-0061-3)
Supplement: Additional file 2: — Questionnaire for practitioners in the dental clinic. [file 12903_2015_61_MOESM2_ESM.docx]

**SN: __________________________ Region: _______________________**

**District: ______________________ Hospital: ______________________**

**This section should be answered by all dental practitioners working in the facility.**

1. Highest professional qualifications:

| 1. HA | 1. DT | 1. ADO | 1. DDS | 1. Others:_____________ |
| --- | --- | --- | --- | --- |

2. On a scale of **0-10**, where **“0”** is **“not comfortable at all”** and **“10”** is **“extremely comfortable”**, how do you rate your competency if performing the following services and restorations using the following materials?

| **Dental Material** | **Perceived competency** |
| --- | --- |
| 1. Tooth extraction |  |
| 1. Dental amalgam restoration |  |
| 1. Conventional resin composite (light cure) |  |
| 1. Packable resin composite (cold cure) |  |
| 1. Glass Ionomer cement |  |
| 1. Zinc Oxide/ Zinc phosphate |  |
| 1. Calcium hydroxide application |  |

3. Do you have the following items in your facility and what is their status?

| **Item** | **Availability of utilities when you require them in order to work** | | | | |
| --- | --- | --- | --- | --- | --- |
|  | **Not available(0)** | **Rarely available (1)** | **Sometimes (2)** | **Most of the time (3)** | **Always (4)** |
| 1. Electricity |  |  |  |  |  |
| 1. Tap water |  |  |  |  |  |

4. On a scale of **0-10**, whereby **“0”** is **“highly unfavourable”** and **“10”** is **“highly favourable”**, how do you generally rate the availability of utilities towards enabling you to carry out restorative services? ___________________

5. In the last 1 month, have you:

1. Performed an extraction 1. Yes 2. No
2. Performed any kind of filling 1. Yes 2. No
